# Few women receive a specific explanation of a stillbirth - an online survey of women’s perceptions and thoughts about the cause of their baby’s death

**DOI:** 10.1186/s12884-019-2289-4

**Published:** 2019-04-26

**Authors:** Berit Höglund, Ingela Rådestad, Ingegerd Hildingsson

**Affiliations:** 1 Department of Women’s and Children’s Health, Uppsala University, 751 85 Uppsala, Sweden; 2 Sophiahemmet University, Stockholm, Sweden; 3 Department of Nursing, Mid Sweden University, Sundsvall, Sweden

## Abstract

**Background:**

In Sweden, three to four out of every 1000 pregnancies end in stillbirth each year. The aim of this study was to investigate whether women who had experienced stillbirth perceived that they had received an explanation of the death and whether they believed that healthcare professionals were responsible for the death of the baby.

**Methods:**

An online survey of 356 women in Sweden who had experienced a stillbirth from January 2010 to April 2014. A mixed-methods approach with qualitative content analysis was used to examine the women’s responses.

**Results:**

Nearly half of the women (48.6%) reported that they had not received any explanation as to why their babies had died. Of the women who reported that they had received an explanation, 84 (23.6%) had a specific explanation, and 99 (27.8%) had a vague explanation. In total, 73 (30.0%) of the 243 women who answered the question “Do you believe that healthcare personnel were responsible for the stillbirth?” stated Yes. The women reported that the healthcare staff had not acknowledged their intuition that the pregnancy was proceeding poorly. Furthermore, they perceived that the staff met them with nonchalance and arrogance. Additionally, the midwife had ignored or normalised the symptoms that could indicate that their pregnancy was proceeding poorly. Some women added that neglect and avoidance among the healthcare staff could have led to a lack of monitoring, which could have been crucial for the outcome of the pregnancy.

**Conclusions:**

Half of the women surveyed reported that they had not received an explanation of their baby’s death, and more than one-fourth held healthcare professionals responsible for the death.

## Background

Stillbirth affects women during their lifetime. In 2016, 432 (3.6/1000) children were born dead in Sweden; two-thirds of these deaths occurred after week 33 of gestation, and approximately 90% occurred before labour started [1]. Ascertaining the exact cause of death is not only important to parents but also critical towards gaining essential information for future pregnancies [2–4]. If the cause of death is known, it is possible to prevent stillbirths in subsequent pregnancies. However, on a case-by-case basis, it can be difficult to determine the exact cause of the baby’s death, even if an autopsy is performed [2, 3].

In Sweden, stillbirth is defined as the death of a baby in utero after 22 completed gestational weeks [1]. Causes of stillbirth vary with gestational age. In a Swedish study (2014), higher proportions of placental abruption; preeclampsia or hypertension; malformations or chromosomal abnormalities; and intrauterine growth restriction or placental insufficiency were more often identified in preterm stillbirths than in term and postterm stillbirths. The latter had higher proportions of umbilical cord complications, birth hypoxia and infections. Infections were more common in postterm (46.5%) than term stillbirths (19.8%) [5]. Flenady et al. (2011) listed overweight, obesity, maternal age and smoking as major risk factors of stillbirth in populations of high-income countries [6].

In a study of 161 singleton pregnancies that ended in stillbirth in the UK, more than half of women whose babies had died in utero suspected that the pregnancy was proceeding poorly and reported reduced or absent foetal movements [7]. In Sweden, according to national guidelines, pregnant women who perceive reduced or absent foetal movements should be offered immediate care at a health facility to assess the condition of the foetus [8].

Although studies conducted in high-income countries (Australia, New Zealand, Sweden, the UK and the US) have shown that sleeping on one’s back during the third trimester is a risk factor for stillbirth [7–11] and although Warland et al. (2015) reported that 56% of women perceived that stillbirth occurred at night [9], the cause of death remains unknown in a large number of cases [10].

### Aim

The aim was to investigate whether women of a stillborn baby perceived that they had received an explanation of the death and whether they believed that healthcare professionals were responsible for the death of the baby.

## Methods

### Design

A mixed-methods design involving an online survey of women who had experienced stillbirth.

### Sample

From the original survey, noneligible responses were deleted prior to the analysis (births before 2010, birth gestational week< 28). The sample consisted of women with self-reported stillbirths after 28 weeks of gestation in the period between January 2010 and April 2014.

### Data collection

Data were collected through an online survey provided by the company Impera in Sweden; the survey was hosted by the Swedish National Infant Foundation [11] and placed on the foundation’s homepage. The foundation supports parents after stillbirth and is a member organisation of the International Stillbirth Alliance (ISA). The participants were self-recruited after being informed about the study through newspapers, Facebook, and newsletters within the organisation. The approach for data gathering was a mixed-methods design, and this online survey covered all of Sweden.

The comprehensive online questionnaire contained 87 questions and addressed respondents’ experiences of foetal movements preceding stillbirth, women’s sleeping positions late in pregnancy and lifestyle factors [12]. Four of the questions included in the survey were used in the present study: “Did you receive an explanation of the baby’s death?” with possible responses of “No, no explanation”, “Yes, but not a specific explanation” and “Yes, a specific explanation”. In addition, two open questions (i.e., “What explanation did you receive for the baby’s death” and “What are your thoughts about why the stillbirth occurred?”) were used. The final question—“Do you believe that healthcare personnel were responsible for the stillbirth?” - allowed only yes or no responses. No personal background data were collected.

The study-specific questionnaire was developed based on a previous survey and clinical experience. The questions have been tested for face-to-face validity.

The study was approved by the Regional Ethical Review Board in Stockholm, Sweden (Dnr. 2011/330–31/3), which implied that individual written informed consent from participants was unnecessary.

### Analysis

Descriptive statistics were used to present numeric data. The qualitative content analysis [13, 14] of the responses to the open-ended questions started with several readings of each response to form an understanding of the text as a whole. Next, the text was divided into five domains from which specific topics were identified and meaning units were extracted (i.e., words, sentences or paragraphs with similar content). Thus condensed, the text became manageable without any loss of its primary content. The abstracted condensed meaning units were assigned codes to identify meaningful categories and themes. Subsequently, codes with similar content were grouped into several subcategories and sorted into broader categories to describe the meaning. The process was performed both forwards and backwards several times with discussions about the analysis, and the content was reflected on until consensus was reached. An example extract from the content analysis appears in Table 1.

**Table 1:** Extract from content analysis

Meaning unit	Condensed meaning unit	Code	Subcategory	Category
The placenta abrupted with the umbilical cord wrapped around the neck and with no visible amniotic fluid	Placenta abrupted with umbilical cord wrapped around the neck; no visible amniotic fluid	Abrupted placenta with wrapped umbilical cord and no amniotic fluid	Abrupted placenta, wrapped umbilical cord with no amniotic fluid	Placental, umbilical cord and amniotic complications

## Results

Of the 356 women in the sample, 173 (48.6%) reported that they did not receive any explanation of the exact cause of the stillbirth, 84 (23.6%) reported receiving a specific explanation, and 99 (27.8%) reported receiving a vague explanation. In an inexact explanation, the woman used the words probably, presumably and likely, while a specific explanation had a clear indubitable message. The years of the stillbirths ranged from 2010 to 2014.

### Explanation of the cause of death

Of the 116 women who reported receiving an explanation of the cause of death, 47 (40.5%) reported explanations of placental complications, including clots, placental abruption, calcifications, a small or immature placenta and vasa praevia. Nearly half (*n* = 20, 43%) of the reported placental complications were abruptions. In contrast, 35 women (30.2%) reported explanations of umbilical cord complications: the umbilical cord was wrapped tightly around the neck or body, or a knot had formed in the cord, which caused hypoxia. Among other explanations, one woman (0.9%) reported obstetric conditions (i.e., growth restriction), 10 (8.6%) reported that the explanation received was ‘sudden infant death syndrome’, nine (6.9%) reported abnormalities, seven (6%) reported infections, one (0.9%) reported uterine rupture, and one (0.9%) reported dystocia. Six women (5.2%) reported not receiving an explanation or that they were waiting for one. A detailed overview of the explanations of stillbirth received is presented in Table 2.

**Table 2:** Explanations from the healthcare personnel for the death of the foetus

1. Placental complications (40.5%)	Clot(s) in placenta and umbilical cord Clots in uterus Placental abruption Calcifications Small or immature placenta Vasa previa
2. Umbilical cord complications (30.2%)	Umbilical cord wrapped tightly around the neck/body Knot in the umbilical cord leading to hypoxia
3. Obstetric conditions (0.9%)	Growth restriction (small for gestational age)
4. Sudden infant death syndrome, SIDS (8.6%)	SIDS
5. Abnormalities (6.9%)	Abnormality/undeveloped organs Down’s syndrome Damaged lungs
6. Infections (6.0%)	Sepsis Infection Infection in the placenta Inflamed placenta GBS (beta-haemolytic streptococci)
7. Birth complications (1.8%)	Rupture of the uterus Dystocia
8. Unknown reasons (5.2%)	Unclear/unknown (still waiting)

### Women’s thoughts about the cause of their baby’s death

The majority of women (*n* = 199, 56%) reported their thoughts on what might have caused their baby’s death. On the basis of these thoughts, seven categories were established, as presented below. The details of the women’s thoughts and explanations are presented in Table 3.

**Table 3:** The women’s thoughts and explanations for the death of the foetus

1. Placental, umbilical cord and amniotic complications (28.6%)	Malnutrition Placenta Ablatio placenta Placenta insufficiency Placenta - less function Placenta - infarction Placenta - calcifications Placenta - small Placenta - clots Umbilical cord Umbilical cord - less flow Umbilical cord - clamped Umbilical cord - knot Umbilical cord - short Umbilical cord - clot Umbilical cord - around the neck/body Amniotic fluid - none left Amniotic fluid - too little Amniotic fluid - too much Pressure changes due to amniotic flow
2. Diseases and complications in the foetus (18.1%)	Arrhythmia of the foetus Cerebral haemorrhage of the foetus Congenital weakness of the foetus SIDS Abnormality Hypoxia Stress in foetus Suffocation (due to contractions) Anaemia/bled to death
3. Risk factors, diseases and infections in mothers (14,1%)	Primipara In vitro fertilisation Large for gestational age Disease in mother Advanced age of the mother Heredity Overweight mother Infection in mother Infection sepsis Urinary infection
4. Pregnancy complications (14.6%)	Gestational diabetes - undiagnosed Gestational hepatosis High blood pressure Incipient preeclampsia Flattening growth curve Estimated due date (birth) - wrong date Postterm pregnancy > 42 + 0 weeks Labour that was delayed/too (< 42 + 0 weeks) Clamped belly Cramps during pregnancy Strong/multiple contractions
5. Tobacco and drugs (3.5%)	Nicotine (smoke and snuff) Drugs Drugs - asthma Drugs - immunosuppressive Drugs - cough drops Drugs - antibiotic Drugs - antiemetic Drugs - pain relief Drugs - vaccination (influenza)
6. External factors (21.6%)	Lack of rest Flights - many at work Slept on wrong side Stress in mother Traffic accident one week before labour Heavy workload It was meant to happen/fate Lack of management Loss of additional controls Lack of responsiveness from healthcare personnel Lack of controls Ultrasound exam was denied Does not trust the healthcare provider anymore - ignorance
7. Unknown reasons (19.1%)	Does not know No opinion No answer yet No cause of death None No idea Many thoughts but I do not know I have no explanation of my own

#### Placental and umbilical cord complications and external factors

The most common category encompassed placental, umbilical cord and amniotic complications. Women who experienced placental and umbilical cord complications reported a dysfunctional placenta, whereas others stated that they had suffered from placental abruption. Some women reported signs of abruption including a hard uterus, bleeding, cold sweats and back pain, although others had shown no symptoms whatsoever. Some women expressed satisfaction with having a specific explanation of the death, whereas others expressed frustration and wanted further explanation as to why the placenta had detached. One woman said, “The placenta had become detached, and he [the foetus] received no blood”. Another stated, “The placenta had lots of clots, so one-third of it did not work, and the baby could not get enough nutrition from the placenta”.

Women reported that deficiencies in antenatal clinical routines during pregnancy constituted an external factor in the stillbirth. For example, negligence in monitoring high blood pressure prevented check-ups of the baby’s condition. Another woman indicated that the baby was too large due to a wrong ultrasound-based estimation and that the obstetricians had advised against caesarean section. One woman stated the following:

“If the midwife hadn’t ignored my high blood pressure, then the baby would have been checked, and they probably would have seen that he wasn’t doing well, since I had only 7 mL of amniotic fluid left when he died”.

Women reported being concerned about medications that could have caused the baby’s death. Others believed that their use of nicotine caused the death:

“Since I used snuff during pregnancy, of course I suspect that it might have been a contributing factor. I also have back problems, for which I’ve been taking painkillers, which could have also been a cause. However, according to the doctor, that shouldn’t have affected… [the stillbirth]”.

Other women reported that sleeping on the wrong side could have been a factor because they had read in newspapers that certain sleeping positions could affect foetuses and pregnancies. Another reason reported was extraordinary stress during pregnancy, and some women mentioned that their jobs had strained their pregnancies. Other concerns related to circumstances, such as squeezing the belly too vigorously during pregnancy. One woman thought that her car accident a week prior to the stillbirth had influenced the outcome of the pregnancy. Another reported believing that uterine death was meant to happen; although she knew the cause of death, she chose to attribute it to fate:

“Now that I know [the reason], I want to believe the explanation that my baby died because of clots in the placenta. A poor but fuzzier explanation I’ve considered is that it was supposed to happen, due to fate because my partner and I were not mature or something or because I was so engaged in my work and stressed by it or because our family situation caused me to not pay enough attention to my body.”

Some women attributed their stillbirths to a postterm pregnancy. One of them had several additional check-ups during the last weeks of pregnancy, whereas another had no additional check-ups. One woman reported the following:

“I think she [the foetus] was in the uterus for too long. The routine ultrasound was delayed by 2 weeks, and the pregnancy was 2 weeks longer than estimated. She [the stillborn baby] was delivered a month after my first estimated date of delivery.”

The women also attributed high-risk pregnancies to gestational hepatosis, vaccinations, diabetes and too much amniotic fluid. As one woman recounted,

“I’m almost certain that I had gestational diabetes, which caused his [the foetus’s] death. I had too much amniotic fluid, and the baby was larger than he should have been at week 35 of gestation.”

### Women’s opinions on healthcare professionals’ responsibility for their baby’s death

Altogether, 243 of the 356 women (68.2%) responded to the statement “Do you believe that healthcare personnel were responsible for the baby’s death?”. Of these women, 170/243 (70%) stated “No,” and 73/243 stated “Yes”.

The women who believed that healthcare personnel were responsible for the deaths often reported that the healthcare staff had ignored their intuition that the pregnancy was proceeding poorly, and some of them believed that they were denied to have an extra ultrasound examination or an induction of labour.

Women described knowing instinctively that something was wrong with the baby, given reduced foetal movements or their own anxiety. One woman reported visiting the labour ward due to a feeling of discomfort one week before the stillbirth. Despite signs that the baby was unwell, the woman’s concern was not taken seriously, and she was sent home from the labour ward:

“I complained several times about my concern, but nobody took me seriously. I visited the labour ward due to reduced foetal movements a week before my son died. The heartbeat was 180 [bpm]. Although the baby was completely still, they did not perform an ultrasound examination. They just sent me home with painkillers.”

The women also mentioned the ignorance and arrogance of the healthcare staff and that midwives had ignored symptoms or normalised them. The women believed that such neglect and avoidance might have prevented the staff from monitoring the pregnancy, which, if monitoring had been performed, could have been crucial to the outcome. As one woman recalled,

“My midwife knew about my complications and concerns but dismissed everything as normal. My son would be alive today if they had performed an ultrasound exam a week before his death.”

## Discussion

Nearly half of the women in the sample reported not receiving any explanation about the cause of their baby’s death, and more than one-fourth held healthcare professionals responsible for the deaths. The women’s thoughts about the causes of death revealed a variety of explanations.

Approximately one-fourth of the women perceived that they received a specific explanation about the underlying cause of their stillbirth. This result aligns with the findings of Smith and Fretts (2007), who reported that specific explanations of intrauterine death are rare [15]. The low percentage of women who perceived that they had received a specific explanation of their baby’s death could be due to the point in time when the healthcare staff provided information about the death. If women received such information during the acute phase directly after stillbirth, then they might have struggled to incorporate that information [16]. The results of the present study underscore the importance of repeating such information, as well as that of the presentation of the information regarding the cause of death insofar as providing both written and verbal information can clarify the diagnosis. Similar to the results of previous studies [3, 4], the explanations received by the women in this study included placental or umbilical cord complications, infections and abnormalities.

The analysis in the present study indicates that some women sensed that their pregnancies were troubled before foetal death in utero was confirmed, as the results of Warland et al. (2015) have also shown [9]. Furthermore, Erlandsson et al. (2012) reported that women described feelings that something was wrong before they sought medical care and received information about foetal death in utero [17]. Women in Trulsson and Rådestad’s (2004) study also reported experiencing a premonition that the pregnancy was dysfunctional before they learned about their unborn baby’s death in utero; in particular, they described symptoms such as decreased or lack of foetal movement and a feeling of heaviness in the stomach [18].

Women in the present sample reported concerns that neglect and avoidance among healthcare professionals could have prompted the lack of check-ups and later influenced the pregnancy outcome. Some reported that healthcare professionals sought to reassure them by stating “It’s nothing to worry about” or “Everything is quite normal”, even though the women felt strongly that something was indeed wrong with their pregnancies. Similar to these results, Trulsson and Rådestad’s (2004) findings indicated that women expressed concerns about being advised to wait and being told that their symptoms were normal [18].

Most women who responded to the survey did not blame healthcare professionals for their stillbirths. However, in several cases, they highlighted that they had not received any information about the cause of death. In some cases, they doubted whether it was the hospital’s fault. One woman reported that it was not the hospital’s fault when the placenta detached but that had the midwife acted otherwise, the baby’s death might have been prevented. Previous research has demonstrated dissatisfaction with healthcare received during pregnancy among women who suffer from stillbirth either before or during delivery [4]. To facilitate good encounters between midwives and expectant mothers, it is therefore important to develop and maintain mutual trust, as well as for midwives to listen to women who report decreased foetal movement or sense that something is wrong with the pregnancy. Researchers have previously concluded that pregnant women seek consistent information about decreased foetal movements and expect responses to their concerns to involve rapid, satisfactory care [19]. In Sweden, the National Board of Health and Welfare (2016) has developed guidelines regarding decreased foetal movements after week 24 of gestation [8]. Such recommendations emphasise the importance of taking women seriously and of providing immediate care when they report reduced or absent foetal movements, largely in light of correlations between perceptions of decreased foetal movements and stillbirth [15].

### Strengths and limitations

Data were collected via an online survey, which made it possible for women of various socioeconomic statuses and ethnicities across Sweden to participate in the study. Furthermore, the survey afforded respondents unlimited space to respond to open-ended questions. However, given the unstructured nature of the data collection, several methodological problems were possible. To obtain more robust data, the data collection was restricted to five years to minimise recall bias. However, these data represent only a proportion of the stillbirths that occurred during that time frame in Sweden, and the participants may not be representative of the true population. In some women, intrauterine death had occurred only a few days before they responded to the survey, whereas for others, it had occurred up to 4 years prior. Such a range allowed reflection periods of various lengths since the time of the stillbirth, which might have influenced the women’s survey responses. Notably, this effect is possible, since parents of more recent stillbirths may not have had/been able to digest all of the information by the time of the survey, and their experiences might have been affected. However, the women’s responses to stillbirths correspond well with those reported in other studies [20]. The sample of 356 women reflects only the total number of stillbirths during the study period. We lack information about background data, such as age or parity, because the survey was anonymous.

We have no access to or information from the hospitals where the women with stillbirths were taken care of. Caregivers might have given other explanations to the women about the cause of their baby’s death. However, in this study, we investigated how the women interpreted the given information. One example of this is the notion of “SIDS”, which is not an accurate definition in terms of stillbirth. Another issue is the lack of information about the women’s sociodemographic status or ethnicity. Their reports could have also been influenced by whether they had given birth later because subsequent pregnancies tend to be managed differently, with serial ultrasound monitoring and the early induction of labour, which, in being perceived as standard care, might have suggested that the index pregnancy was mishandled. Finally, the question “Do you believe that healthcare personnel were responsible for your baby’s death?” allowed only two responses (i.e., *Yes* or *No*), which limited the capacity to identify women who were unsure.

## Conclusion

Nearly half of the women perceived that they did not receive any explanation about the exact cause of the stillbirth, and more than one-fourth of the women held healthcare professionals responsible for the death. Some women reported sensing that their pregnancies were proceeding poorly but that staff members did not take their concerns seriously. Caregivers need to be sensitive to women’s concerns and clearly frame their messages to avoid misinterpretations when they give information about the cause of stillbirth.
